# Supplementary material for: Enhanced Desulfurization by Tannin Extract Absorption Assisted by Binuclear Sulfonated Phthalocyanine Cobalt Polymer: Performance and Mechanism
Source: Materials (Basel). 2023 Mar 15;16(6):2343. doi: 10.3390/ma16062343 (PMC10057467; doi:10.3390/ma16062343)
Supplement: Supplementary file 1 [file materials-16-02343-s001.zip › materials-2174765-supplementary.pdf]

1. Experimental system

The experimental system can be divided into four main bodies: simulated gas distribution system, reaction system, tail gas absorption unit and gas detection system.The flow chart of the whole experimental system is shown in the figure below.The gas distribution system of simulated flue gas is composed of 99.99%N_2 cylinder gas and 99.50%H_2S cylinder gas. Each cylinder is equipped with corresponding pressure reducing valve.The two kinds of gas were controlled by the corresponding mass flowmeter and digital display instrument to obtain a certain concentration of simulated flue gas, which was mixed into the gas mixing tank, and then passed into the three-port flask to be absorbed by the extract desulfurizer.The simulated flue gas after the reaction was dehydrated by drying tube and then entered gas chromatograph for analysis.Finally, the exhaust gas absorption bottle is used to remove the incomplete absorption of gas pollutants.

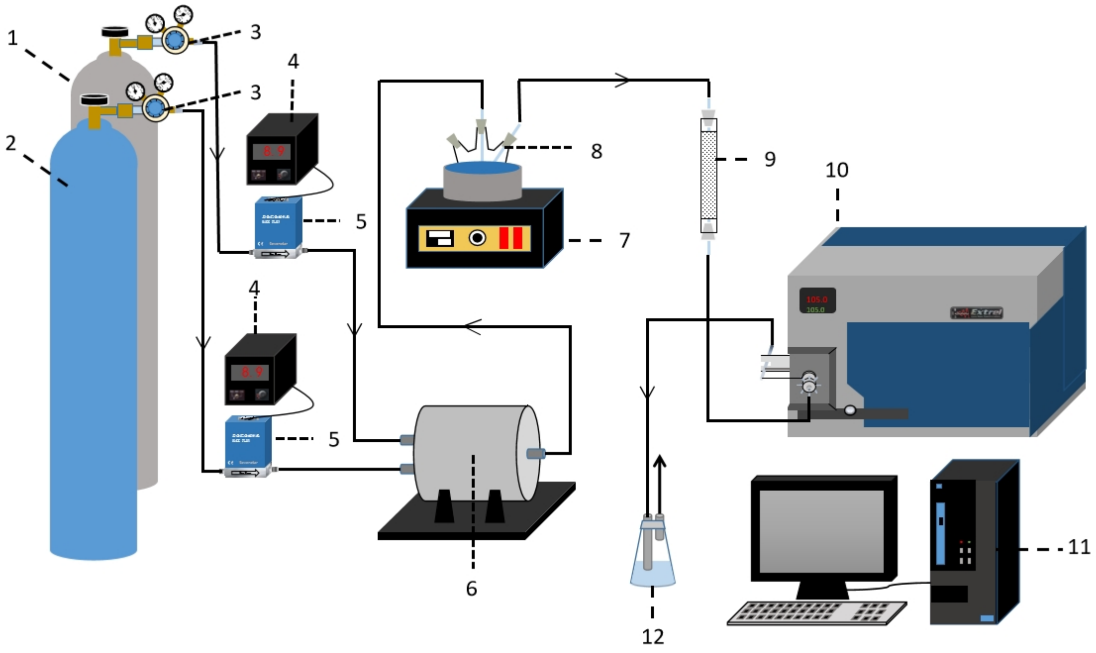

Figure S1. Flow chart of the experimental system (1 N_2 cylinders; 2 H_2S cylinders; 3, relief valve; 4, digital display instrument;5, mass flow controllers; 6, gas mixing tank; 7, thermostatic magnetic stirrer; 8, three-necked flask; 9, drying tube; 10, Gas chromatograph; 11, Data analysis workstation; 12, tail gas treatment system）.

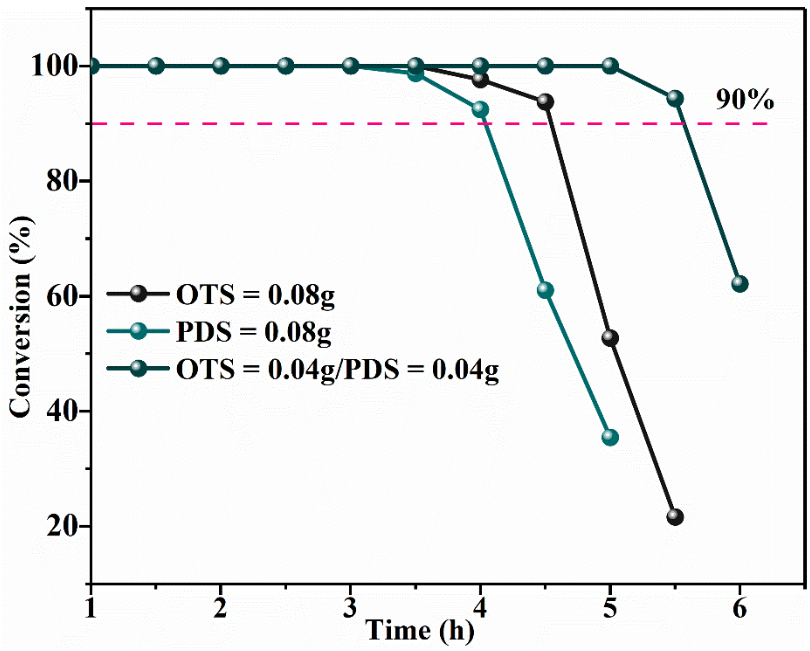

Figure S2. The conversion curve of H_2S removal with the addition of OTS and PDS singly or simultaneously without TE addition.
